# Application of the *FLP/LoxP-FRT* recombination system to switch the eGFP expression in a model prokaryote

**DOI:** 10.1515/biol-2022-0019

**Published:** 2022-03-11

**Authors:** Junhao Dan, Huafeng Deng, Yumei Xia, Yijie Zhan, Ning Tang, Yao Wang, Mengliang Cao

**Affiliations:** Longping Branch of Graduate School, Hunan University, No. 2 Lushan South Road, Yuelu District, Changsha, Hunan Province 410082, People’s Republic of China; State Key Laboratory of Hybrid Rice, Hunan Hybrid Rice Research Center, No. 736 Yuanda Road, Furong District, Changsha, Hunan Province 410125, People’s Republic of China

**Keywords:** *FLP/LoxP-FRT*, gene switch, *Escherichia coli*, *eGFP*, prokaryotic

## Abstract

In prokaryotes, few studies have applied the *flippase* (*FLP*)/P1-flippase recombination target (*LoxP-FRT*) recombination system to switch gene expression. This study developed a new method for switching gene expression by constructing an *FLP*/*LoxP-FRT* site-specific recombination system in *Escherichia coli*. To this end, we placed the *Nos* terminator flanked by a pair of *LoxP-FRT* in front of enhanced green fluorescent protein (*eGFP*). The *Nos* terminator was used to block the expression of the *eGFP*. When a plasmid expressing *FLP* was available, deletion of the *Nos* terminator would allow expression of *eGFP*. The regulatory effect was demonstrated by *eGFP* expression. The efficiency of the gene switch was calculated as high as 89.67%. The results showed that the *FLP*/*LoxP-FRT* recombinase system could be used as a gene switch to regulate gene expression in prokaryotes. This new method for switching gene expression could simplify the gene function analysis in *E. coli* and other prokaryotes, as well as eukaryotes.

## Graphical abstract

**Figure:**
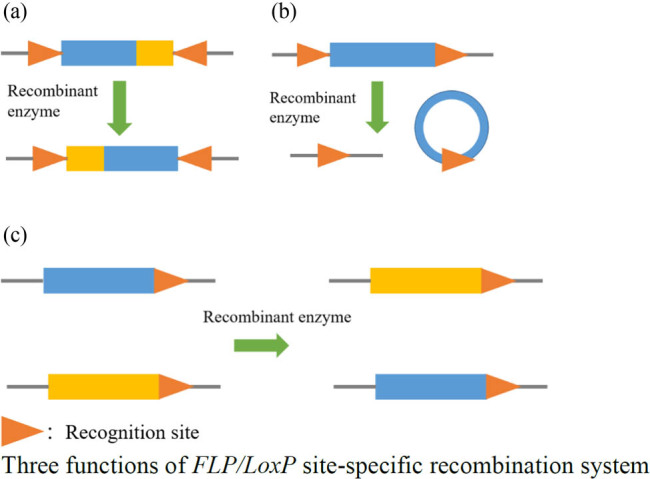

## 1 Introduction

*Escherichia coli* is a Gram-negative bacterium. Most *E. coli* strains have a capsular structure and fimbriae. Due to its simple structure and well-understood genetic background, it is an important model organism and one of the most commonly used bacteria for gene transformations currently [1]. Terminator is a DNA sequence that functions to terminate DNA transcription and release RNA. Hence, the terminator plays an important role in regulating gene expression [2,3]. At present, some universal terminators are used in plant genetic transformation vectors and most of them are derived from viruses or other microorganisms. Among them, *Nos* terminator is one of the most widely used terminators in plant molecular breeding [4].

The flippase (*FLP*) recombinase system is derived from the 2 µm plasmid of *Saccharomyces cerevisiae*. Depending on the location and direction of the flippase recombination target (*FRT*) recognition sites, *FLP* recombinase can invert, recombine, and position the DNA sequence between the recognition sites [5]. When *FRT* recognition sites have different orientations and are located on the same chromosome, *FLP* recombinase inverts the sequences between the recognition sites; when *FRT* recognition sites have the same orientation and are located on the same chromosome, *FLP* recombinase removes the entire region between the recognition sites, leaving only one recognition site; and when *FRT* recognition sites are in the same orientation but are located on different chromosomes, the two chromosomes will exchange DNA around the recognition sites under the action of FLP recombinase [6]. As the *FLP* recombinase system has been shown to have high recombination efficiency and target in eukaryotes, it is widely used in higher eukaryotes such as rice, Arabidopsis and tobacco for deletion of exogenous and marker genes at present [7,8,9,10,11,12]. Studies have shown that in the *FLP* recombination system, gene recombination efficiency was higher when using the locus of crossing over in the P1-flippase recombination target (*LoxP-FRT*) fusion recognition site compared with the *FRT* single recognition site [13,14]. Introduction of a nuclear localization signal (NLS) can also improve gene recombination efficiency [10].

In addition to eukaryotes, the *FLP*/*FRT* recombination system has also been used in prokaryotes. A markerless mutant was generated in cyanobacteria by using *FLP* recombinase to delete the kanamycin (KAN) resistance gene between two *FRT* recognition sites [15]. The *FLP* recombinase was used to eliminate the resistance genes between two *FRT* sites in *E. coli* [16]. Apart from this, there have been few studies about the *FLP*/*LoxP-FRT* site-specific recombination system operating as a gene switch to regulate target gene expression.

The purpose of this study was to develop a new method for switching gene expression by constructing an *FLP*/*LoxP-FRT* site-specific recombination system in *E. coli*. The regulatory effect was demonstrated by enhanced green fluorescent protein (*eGFP*) expression. This new method of switching gene expression could simplify the analysis of the gene function in prokaryotes and eukaryotes. The effectiveness of this method provides a reference for the *FLP*/*LoxP-FRT* recombinase system as a gene switch and basis for the future gene switch of other recombinase systems.

## 2 Materials and methods

### 2.1 Bacterial strains and transformations

*E. coli* strain BL21 was used for this study. The liquid medium used for *E. coli* growth was Luria Broth (LB; 10.0 g tryptone, 5.0 g yeast extract, and 10.0 g sodium chloride per 1 L water), whereas LB agar was used as the solid medium (LB with 15.0 g agar per 1 L). For selective media (used to isolate transformed bacteria), the final concentrations of KAN, ampicillin (AMP), and isopropyl-β-D-thiopyrgalactoside (IPTG) were 50 µg/mL, 100 µg/mL, and 0.5 mmol/L, respectively.

**Figure 1:**
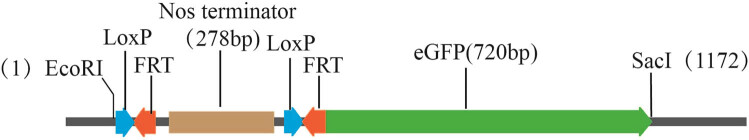
Construction scheme of Ep30TL vector. Using EcoRI and SacI enzymes to double digest the pET-21a (+) vector, the vector Ep30TL was obtained by attaching seq30TL to the pET-21a (+) vector with T4 DNA ligase.

**Figure 2:**
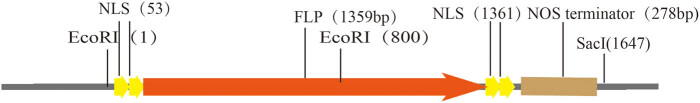
Construction scheme of p30TK vector. Using EcoRI and SacI enzymes to double digest the pRSFDuet-1 vector, the vector p30TK was obtained by attaching seq30TK to the pRSFDuet-1 vector with T4 DNA ligase.

Vectors pET-21a (+) and pRSFDuet-1 were purchased from GenScript Biotechnology Co. Ltd. The Ep30TL vector was constructed using EcoRI and SacI enzymes (Thermo Fisher Scientific, Tokyo, Japan) to double digest the pET-21a (+) vector, and then the synthetic fragment seq30TL (containing *Nos* terminator between two *LoxP*/*FRT* sites and tagged with *eGFP*) was ligated to the pET-21a (+) vector by T4 DNA ligase (Promega, Wisconsin, USA) (Figure 1). The p30TK vector was constructed by double digestion of the pRSFDuet-1 vector with EcoRI and SacI enzymes followed by the ligation of the synthetic fragment seq30TK (containing *FLP* between NLS and tagged with *Nos* terminator) to the pRSFDuet-1 vector by T4 DNA ligase (Figure 2). Ep30TLK competent cells were prepared as described by Sambrook and Russell [17]. The vector p30TK was transformed into Ep30TL competent cells by electroporation and the transformed line (Ep30TLK) was grown at 37℃ with shaking at 180 rpm/min for 1 h. Approximately, 100–200 µL of the resulting culture was spread on LB agar containing KAN + AMP + IPTG.

### 2.2 Vector sequence verification

The following primer sequences were designed for verification to ensure the sequences of the Ep30TL, p30TK, and Ep30TLK vectors were correct. The primers F1 and R1 were used to verify the *Loxp-FRT-Nos-Loxp-FRT-eGFP* vector Ep30TL sequence, where *LoxP-FRT* sequences with the same orientation were added at both ends of the *Nos* terminator, and the *eGFP* gene was added at the 3′ end. The primers F2 and R2 were used to verify the *FLP* recombinase expression vector p30TK. The EcoRI restriction site was introduced at the 5′ end of the *FLP* gene and the SacI restriction site was introduced at the 3′ end. The Ep30TLK vector, the product of p30TK transformation into Ep30TL competent cells by electroporation, was verified by sequencing with F1, R1, F2, and R2 primers. The synthesis and sequencing of the primers were completed by Tsingke Biotechnology Co. Ltd.

F1: CCGGATATAGTTCCTCCTTTC

R1: AGATCTCGATCCCGCGAAAT

F2: GTATATGTGCCTACTAACGC

R2: CTTTCATCAATTGTGGAAGA.

### 2.3 Regulatory efficiency calculations

Ep30TLK was cultured on LB medium supplemented with KAN + AMP + IPTG at 37°C. Samples of 50 cultures of 100 µL each were mounted on glass slides. Ep30TL competent cells were negative control without p30TK. Prepared slides were then analyzed with a Confocal 880 laser, confocal scanning microscope and stimulated with a laser at 488 nm to obtain scanning images of *E. coli* at different points in time post inoculation.

The study has set three replicates to calculate the efficiency of gene switch. The calculations of total bacteria were made from bacterial populations that were more or less similar in number in the negative control and treated bacterial cells. The efficiency of the gene switch was calculated as the number of single green fluorescent colonies divided by the total number of colonies.

DNA extraction was performed according to the instructions of the plasmid DNA microextraction kit (Magen, Guangzhou, China). Gel purification was performed according to the instructions of the gel midi purification kit (Tiangen, Beijing, China). Restriction digests were incubated at 37°C for 30 min according to the manufacturer instructions (Thermo Fisher Scientific, Tokyo, Japan).

### 2.4 Statistical analysis

Preliminary calculations were performed on the data using excel tables, SPSS16.0 statistical analysis software was used to perform variance analysis on the data of deletion efficiency of *FLP* recombinase, and all the data were analyzed by the least significant difference method at 5% level.

## 3 Results and analysis

### 3.1 Verification of transformants with Ep30TL and p30TK

**Figure 3:**
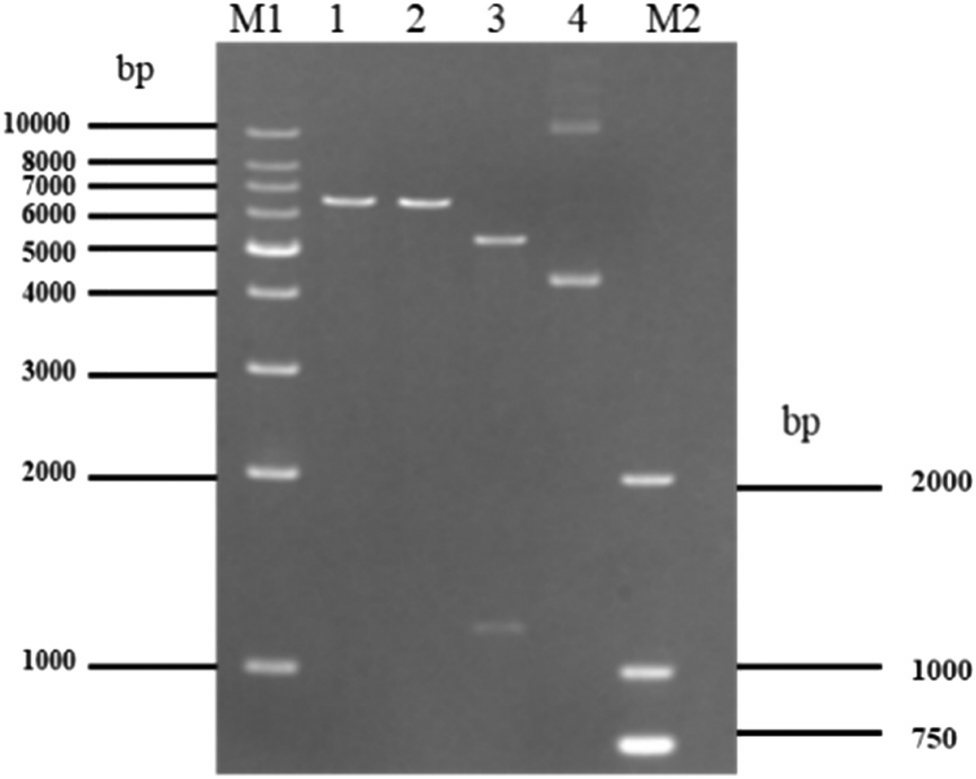
Visualization of Ep30TL plasmid digest products. Lane M1 and M2 are Marker-1kb and Marker-DL2000, respectively (Tiangen, Beijing, China); they showed molecular size marker as indicated alongside in kilodaltons. Lane 1: product of Ep30TL plasmid digested with EcoRI. Lane 2: product of Ep30TL plasmid digested with SacI. Lane 3: product of Ep30TL plasmid digested with EcoRI and SacI. Lane 4: undigested Ep30TL plasmid.

As shown in Figure 3, the Ep30TL plasmid was identified by single and double enzyme digestion with restriction endonucleases, EcoRI and SacI. The single digests each resulted in DNA fragments of 6,613 bp while the double digest resulted in two fragments of 5,441 bp and 1,172 bp, respectively; one was the pET-21a (+) plasmid vector and the other was the seq30TL insertion fragment. The electrophoresis results showed that the expected fragments were obtained and the sequencing results were consistent with the enzyme digestion results, further indicating that the cells were successfully transformed with the Ep30TL plasmid.

**Figure 4:**
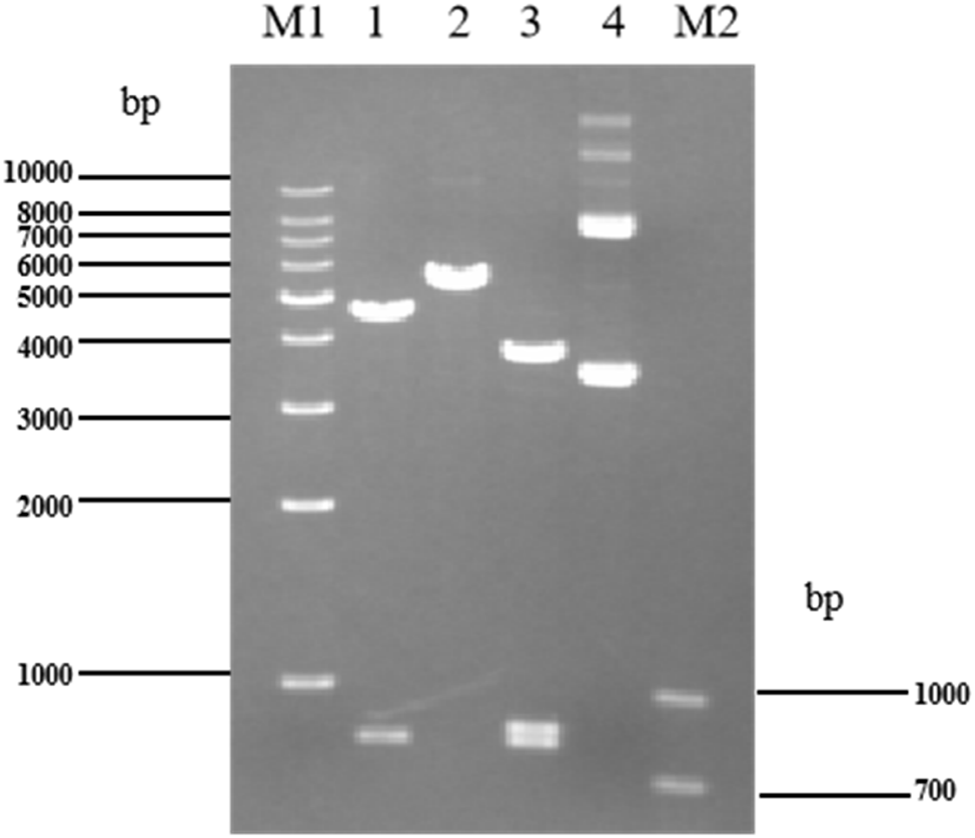
Visualization of p30TK plasmid digest product. Lane M1 and M2 are Marker-1kb and Marker-DL1000, respectively (Tiangen, Beijing, China). Lane 1: product of p30TK plasmid digested with EcoRI. Lane 2: product of p30TK plasmid digested with SacI. Lane 3: product of p30TK plasmid digested with EcoRI and SacI. Lane 4: undigested p30TK plasmid.

The p30TK plasmid was verified using the same method. As shown in Figure 4, the EcoRI enzyme single digest resulted in 4,666 bp and 800 bp fragments, while the SacI enzyme single digest resulted in a fragment of 5,466 bp. Double digest with EcoRI and SacI resulted in fragments of 3,819 bp, 847 bp, and 800 bp, consistent with the expectations. The p30TK plasmid isolated from the transformed bacteria was sequenced and found to be 100% homologous to the reference sequence, thus demonstrating that the transformation with p30TK was successful.

### 3.2 Expression of p30TK in Ep30TL competent cells

Through electroporation method, p30TK was transformed into Ep30TL competent cells, and the transformed product (Ep30TLK) and negative control were added to LB medium separately. Bacterial culture was then sampled to observe the fluorescence with a laser confocal microscope.

**Figure 5:**
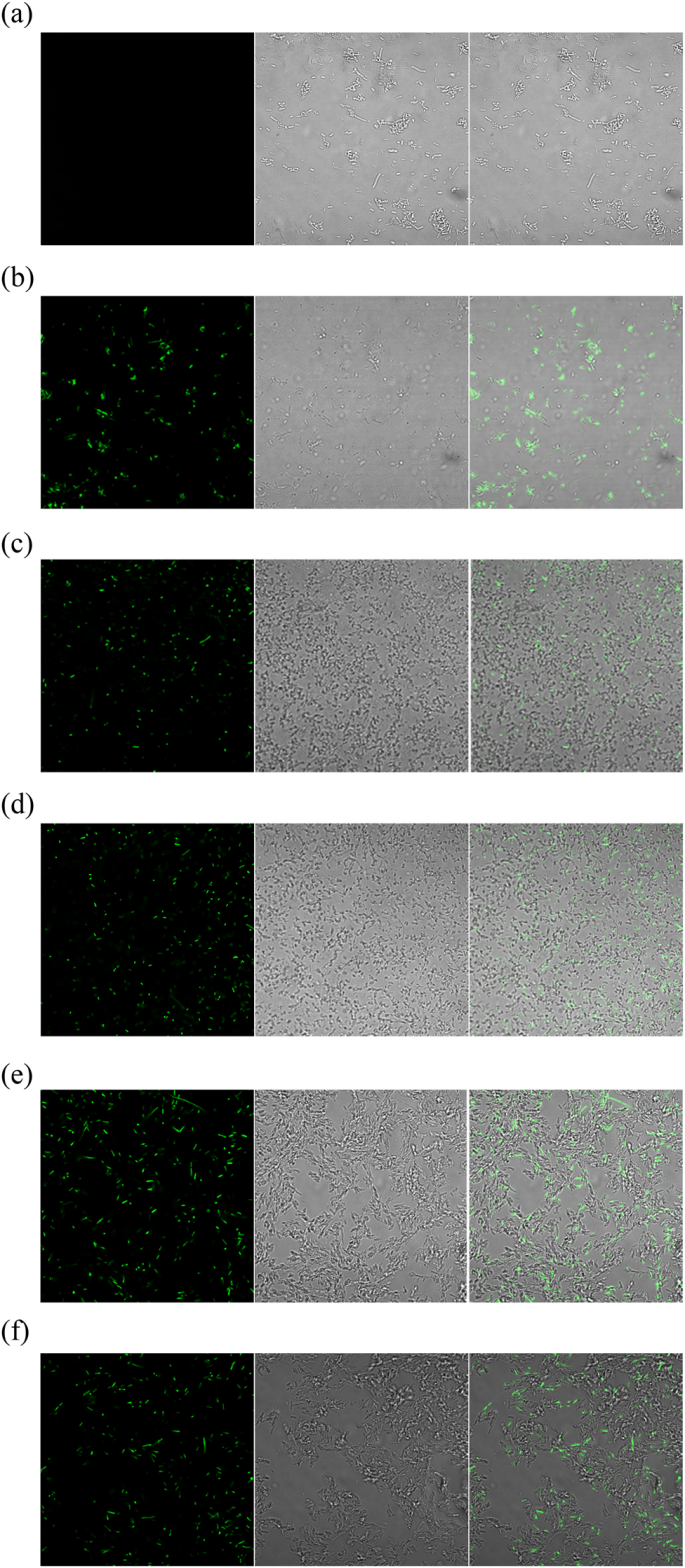
*eGFP* expression in different samples under the laser confocal microscope. Left: Fluorescence images; middle: bright field images; right: overlay images.

As shown in Figure 5, *eGFP* did not express in the negative control (Figure 5a), demonstrating that the gene *eGFP* was locked in Ep30TL. The expression of *eGFP* was observed in Ep30TLK in five different fields (Figure 5b–f), which proved that p30TK had been transformed into Ep30TL competent cells, and the *Nos* terminator was deleted by *FLP* recombinase in Ep30TLK. The *FLP*/*LoxP-FRT* recombination system to switch the *eGFP* expression was successful in *E. coli* by observing the expression of *eGFP*.

### 3.3 Regulatory efficiency of gene switch

**Figure 6:**
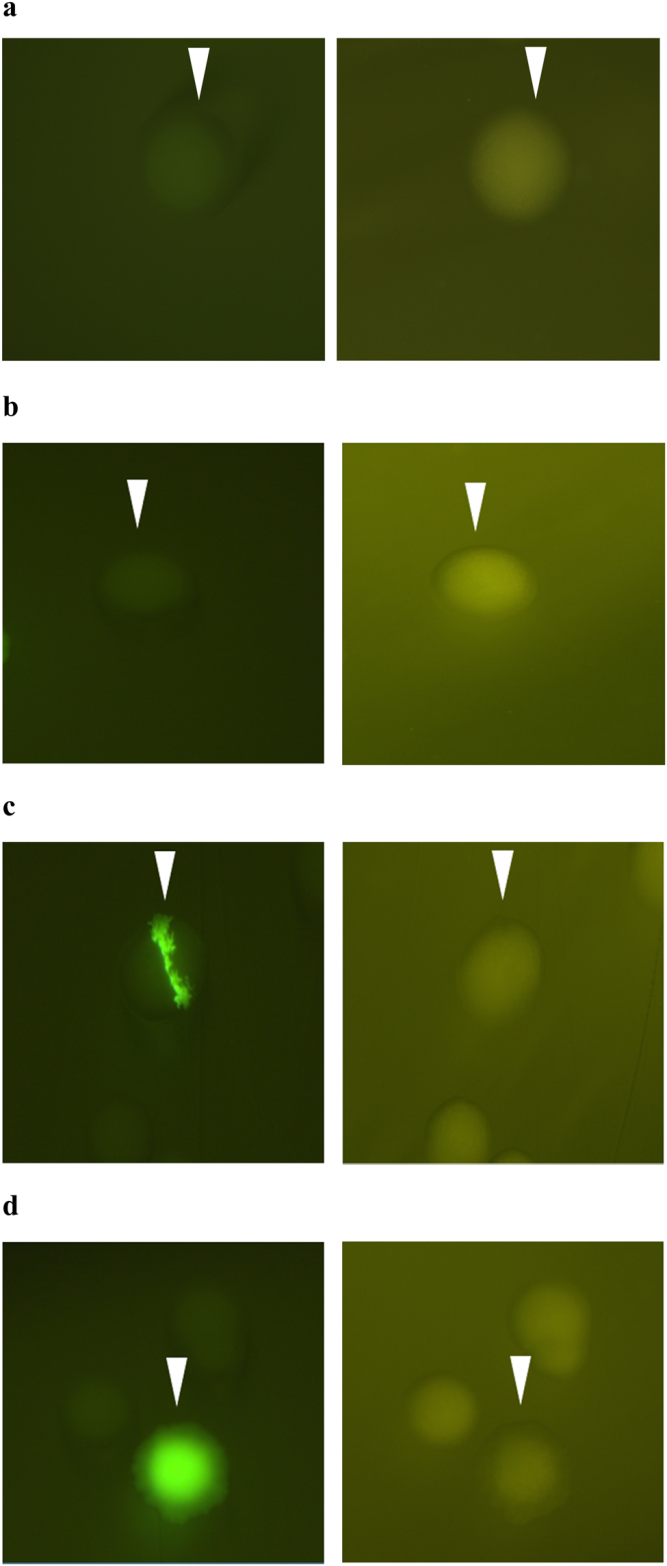
Expression of *eGFP* in single colonies in the transformation of bacterial cells. (a) Single white colony in the negative control. (b) Single white colony in Ep30TLK. (c) Single green and white colony in Ep30TLK. (d) Single green colony in Ep30TLK. The left column shows images taken with green fluorescent microscopy and the right column shows images taken with bright field microscopy.

In the negative control, expression of *eGFP* was not observed in bacterial cells (Figure 6a). In Ep30TLK, expression of *eGFP* was observed in single colonies under a fluorescence microscope, with three different types of colonies being identified: those with no *eGFP* expression (“single white colonies,” Figure 6b); those with some *eGFP* expression (“single green and white colonies,” Figure 6c); and those with full *eGFP* expression (“single green colonies,” Figure 6d). Single white colonies resulted from *Nos* terminator not being deleted, preventing *eGFP* expression. Similarly, the appearance of single green and white colonies was due to the incomplete deletion of *Nos* terminator by *FLP* recombinase, while the occurrence of green fluorescent single colonies was because *eGFP* could be expressed after the complete deletion of *Nos* terminator. Thus, it could be concluded that the *FLP*/*LoxP-FRT* recombination system could switch the gene *eGFP* expression in a single colony.

Then, the regulatory efficiency of gene switch in *E. coli* could be calculated. Statistical results are shown in Table 1; the efficiency of the gene switch in Ep30TLK was calculated in this study as ∼87.10–89.67%.

**Table 1:** Regulatory efficiency of gene switch in *E. coli*

Treatment	The total population of bacteria in each plate	Rate of *FLP* recombinant enzyme deletion (%)
Negative control	315	0
Ep30TLK1	296	87.60 ± 2.04
Ep30TLK2	306	87.10 ± 2.91
Ep30TLK3	284	89.67 ± 1.42

### 3.4 Verification of Ep30TLK plasmid

**Figure 7:**
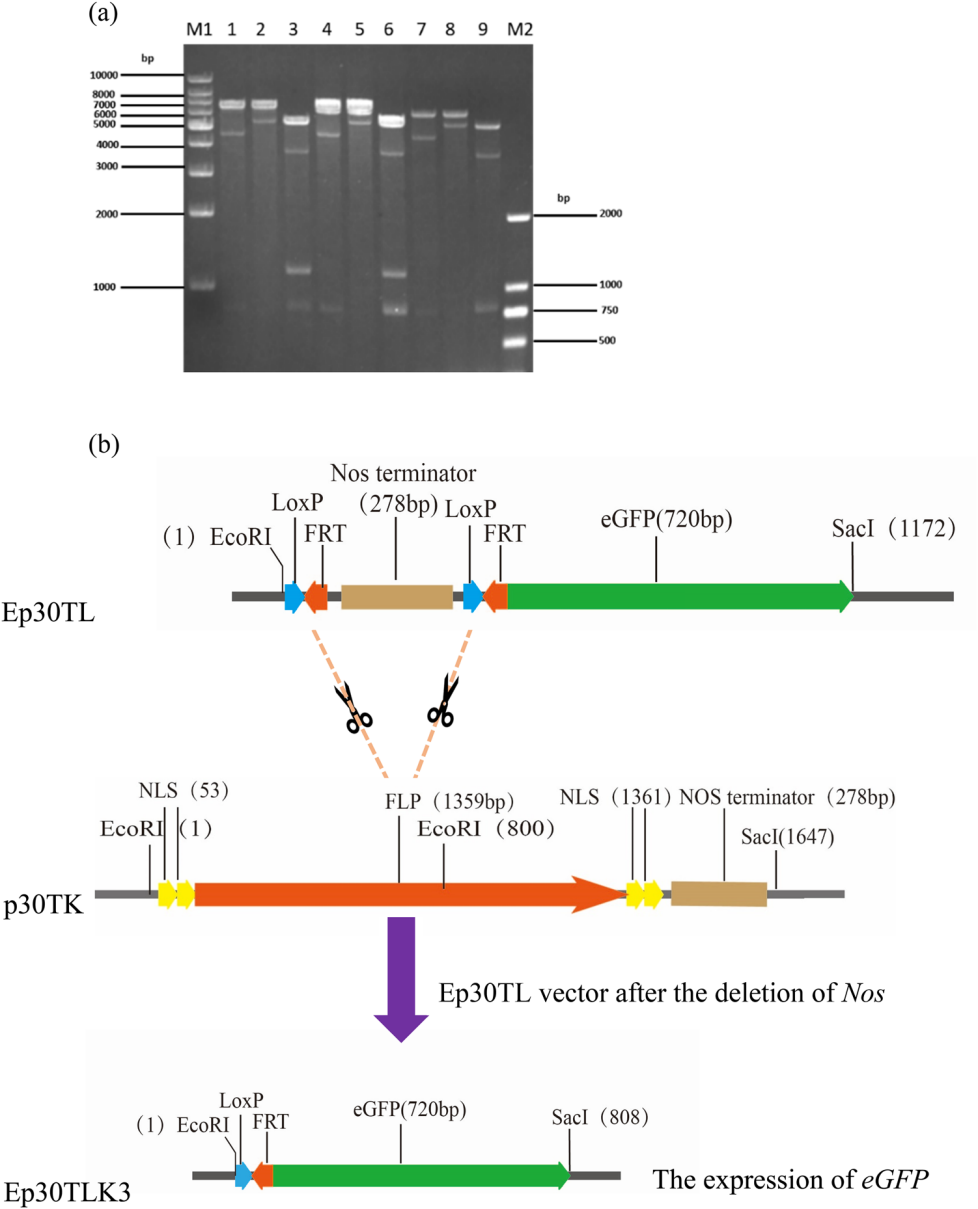
Identification of restriction enzyme digestion of Ep30TLK plasmid. (a) Lane M1 and M2 are Marker-1kb and Marker-DL2000, respectively (Tiangen, Beijing, China). Lanes 1, 2, and 3: products of Ep30TLK1 plasmid digestion with EcoRI, SacI, and EcoRI + SacI, respectively. Lanes 4, 5, and 6: products of Ep30TLK2 plasmid digestion with EcoRI, SacI, and EcoRI + SacI, respectively. Lanes 7, 8, and 9: products of Ep30TLK3 plasmid digestion with EcoRI, SacI, and EcoRI + SacI, respectively. (b) In the Ep30TLK3 plasmid, the deletion of *Nos* in Ep30TL leads to expression of *eGFP*.

The three different single colonies shown in Figure 6 were cultured in liquid medium, then plasmids were extracted from each. Extracted plasmids were named Ep30TLK1, Ep30TLK2, and Ep30TLK3, each corresponding to the white, green and white, and green colonies, respectively. As shown in Figure 7a, EcoRI and SacI double digestion of Ep30TLK1 and Ep30TLK2 results in 1,172 bp fragments; when the same digestion was performed with Ep30TLK3, an 808 bp fragment was seen but not a 1,172 bp fragment, thus indicating that *Nos* terminator in Ep30TLK3 had been deleted by *FLP* recombinase. Results of each plasmid from single digests with EcoRI and SacI were also in line with the expectations. Finally, the Ep30TLK1, Ep30TLK2, and Ep30TLK3 plasmids were sequenced to unambiguously verify their identity and were found to be consistent with the reference sequence.

The double digestion model of vector Ep30TLK is shown in Figure 7b. After p30TK was transformed into Ep30TL competent cells, if *FLP* recombinase fully deleted *Nos* terminator in Ep30TL, the plasmid Ep30TLK3 could be obtained after identification from *eGFP* expression. The deletion of the *Nos* terminator would lead to an 808 bp fragment resulting from double digestion with EcoRI and SacI. The results shown in Figure 5 were consistent with the double digestion model.

## 4 Discussion

In this study, the *FLP*/*LoxP-FRT* recombination system was used as a gene switch, controlling the temporal and spatial specificity to precisely regulate target gene expression. The *FLP* recombination acted as the “gene key” while *Nos* terminator was the “gene lock” that hindered the expression of *eGFP*. When the *FLP* recombinase recognized the *LoxP-FRT* fusion site and deleted the *Nos* terminator, the “gene key” opened the “gene lock” and *eGFP* could be expressed. The efficiency of the gene switch was calculated as high as 89.67%.

Exploring the efficiency of the *FRT*/*LoxP-FRT* recombination system in *E. coli* provides a reference for the research of prokaryotes with other site-specific recombination systems. For example, when studying the *Cre*/*LoxP* system from bacteriophage P1, based on the prior construction of a label-free transgenic *Chlamydomonas reinhardtii* [18], the *Cre* recombinase can be combined with the *LoxP-FRT* fusion site and applied to other prokaryotes. Alternatively, the method presented in this study could be used with the recombinase/recombination site (*R*/*RS*) system from *Zygosaccharomyces* to verify the effectiveness and gene switch efficiency of the *R*/*RS* recombinant systems quickly and efficiently.

This principle has been shown previously using the *Cre*/*loxP* site-specific recombination system as a gene switch in hybrid rice; one cassette was the KEY, containing a nuclear-localized *Cre* recombinase driven by the green-tissue-specific promoter *rbcS,* while another cassette was the LOCK, containing a *Nos* terminator between two *loxP* sites. When the two cassettes were pyramided into hybrid rice, the *Cre* recombination from the KEY would excise *loxP-NosT* in the LOCK, the gene of interest could express in green tissues but not express in the endosperm [19]. For future research in eukaryotes, we can consider changing the “gene key” promoter to regulate gene expression specifically. In the *FLP*/*LoxP-FRT* gene switch, the expression of *FLP* recombinase is designed to be driven by the specific promoter. The design strategy is to first deactivate all gene expressions and then activate expression in only the desired locations.

In addition to the *FLP*/*LoxP-FRT* gene switch that we constructed in this study for switching *eGFP* gene expression in the *E. coli*, we have considered another method for switching gene expression. When the promoter flanks by a pair of *FRT* in the opposite direction (*FRT*-promoter-*FRT* [opposite]), it can drive the expression of the *eGFP*. When *FLP* is expressed, the expression of *eGFP* is hindered because the promoter has been reversed. Compared with the “*FRT*-promoter-*FRT* (opposite)” method, our *FLP*/*LoxP-FRT* gene switch is stable. In the “*FRT*-promoter-*FRT* (opposite)” method, the promoter sequences between the *FRT* sites (opposite) would be inverted constantly and that may not regulate gene expression precisely. Therefore, as shown in our *FLP*/*LoxP-FRT* gene switch, the *Nos* terminator was added between the *LoxP-FRT* sites (same orientation) to hinder the expression of *eGFP*, when a plasmid expressed *FLP* recombinase, the *Nos* terminator was deleted instead of inverted, *eGFP* was expressed, and a stable gene switch was constructed in *E. coli*.

In conclusion, the *FLP*/*LoxP-FRT* gene switch has good development potential in prokaryotes and eukaryotes. First, in the research on the synthesis of natural products by recombinant microorganisms, the *FLP*/*LoxP-FRT* gene switch can be used to dynamically regulate the metabolic flow, that is, when the microorganisms grow to a certain stage, the “gene key” opens the “gene lock” to specifically express a specific protein at a specific time, thereby effectively distributing the intracellular metabolic flux and ultimately increasing the output of natural products. Second, for future research in eukaryotes, such as the cultivation of transgenic plants, the *FRT*/*LoxP-FRT* gene switch has the advantage of the ability to regulate the expression of foreign genes and marker genes to produce marker-free transgenic plants, thereby relieving public concerns about transgenic plants.
